# Universal Transient Dynamics of Electrowetting Droplets

**DOI:** 10.1038/s41598-018-19167-7

**Published:** 2018-01-16

**Authors:** Quoc Vo, Haibin Su, Tuan Tran

**Affiliations:** 1 School of Mechanical and Aerospace Engineering, Nanyang Technological University, 50 Nanyang Avenue, 639798 Singapore, Singapore; 2 Institute of Advanced Studies, Nanyang Technological University, 60 Nanyang View, 639673 Singapore, Singapore

## Abstract

Droplet spreading on substrates by electrowetting exhibits either of the two transient behaviours: one characterised by contact line oscillation, and the other one by slow spreading dynamics. The transition between these behaviours remains elusive due to the current limited understanding of the spreading dynamics on the hydrodynamical and electrical properties of electrowetting systems. To understand this transition we propose a model capturing the transition’s occurrence based on both the hydrodynamical and electrical parameters. We derive the critical viscosity at which the transition occurs and reveal its subtle and often hidden dependence on the electrowetting dynamics. We find and experimentally verify that the condition for minimization of droplets’ actuation time is only achieved at the transition. Particularly, the transition time as a function of damping ratio exhibits the general feature of Kramers’ reaction-rate theory.

## Introduction

**Figure 1:**
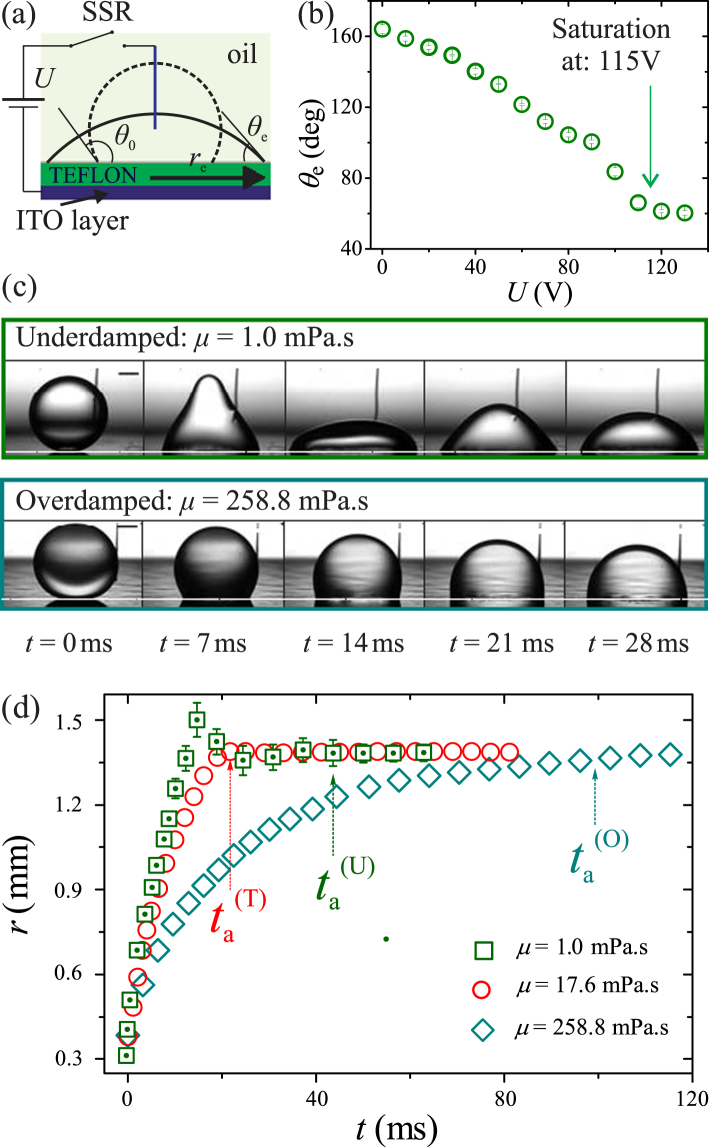
(**a**) Schematic of an EWOD experimental setup. (**b**) Dependence of equilibrium contact angle *θ*_e_ on applied voltage *U* showing that contact line saturation occurs at *U* = 115 V. (**c**) Snapshots showing deformation of an underdamped droplet (upper panel) and an overdamped droplet (lower panel) after a voltage of 100 V is applied. The scale bars represent 0.5 mm. The dashed lines mark the substrate level. (**d**) Spreading radius *r* versus time *t* for underdamped droplets (open squares), overdamped droplets (open diamonds), and droplets with transitional behaviour (open circles).

A droplet resting on a flat electrode changes its contact angle if a voltage difference between the droplet and the electrode is applied. In this so-called electrowetting phenomenon, it is advantageous to electrically insulate the droplet and the electrode to prevent current and the resulting electrolysis^1^. Such setting is termed electrowetting-on-dielectric (EWOD)^2^, and has become increasingly important in diverse applications requiring active control of droplets such as fast response displays^3^, high-power energy harvesting^4,5^, digital microfluidics^6^, liquid lens^7^, light valves^8^, fast optical imaging^9^, optical films^10^, and tissue engineering^11^. In a typical EWOD setup (Fig. 1a), an electrically conductive substrate is coated with a thin insulating layer. A droplet deposited on the insulating layer is in contact with an electrode on top, while the conductive substrate is connected to another electrode. When a voltage *U* is applied between the two electrodes, surface energy at the liquid-solid interface is changed, causing the droplet to deform and take another equilibrium state. The contact angle *θ*_e_ of the droplet at the new equilibrium state can be related to the initial contact angle *θ*_0_ using the well-known Young-Lippmann (Y-L) equation^1,12^ cos *θ*_e_ − cos *θ*_0_ = *εε*_0_*U*^2^/2*dσ* = *η*, where *η* denotes the so-called electrowetting number, *ε*_0_ is permitivity of free space, and *ε*, *d*, *σ* respectively are the dielectric constant, the insulating layer thickness, and the interfacial tension of the droplet’s liquid and the surrounding medium.

While the Y-L equation gives predictions in good accord with the measured changes in contact angles of a droplet under an electric field^1^, it only relates the equilibrated contact angles before and after the electric field is applied. Thus, it cannot be used to describe the transient dynamics between the two equilibrium states. Understanding of droplet characteristics during actuation, however, plays a critical role in applications utilizing EWOD for droplet manipulation. In particular, the relations between the system parameters, e.g., droplet size, liquid properties, applied voltage, and the resulting transient characteristics, e.g., the actuation time, are of both fundamental and engineering interests. The goal of this paper is to investigate these relations experimentally and analytically.

## Results and Discussions

In our experiments, illustrated in Fig. 1a, we use aqueous glycerin solutions consisting of glycerol, DI water, and 0.125 M sodium chloride as working liquids for generating droplets. The electrical conductivity of the solutions of DI water and 0.125 M sodium chloride is ≈8.8 EC. By adjusting the glycerol concentration, we vary the viscosity *μ* of the solutions from 1 mPa · s to 258.8 mPa · s. We generate droplets by dispensing liquid from a micro-needle and vary the droplet radius *R* from 0.05 mm to 1.6 mm. The substrate is made of an indium tin oxide (ITO) glass slide covered by a fluoropolymer layer (Teflon AF-1600, DuPont) having dielectric constant *ε* = 1.93 and thickness *d* = 2.5 μm. The Teflon layer acts as a hydrophobic coating and electrically insulates the ITO layer. We immerse both the droplets and the substrate in silicone oils with viscosity *μ*_o_ varying in the range 1.75 mPa · s ≤ *μ*_o_ ≤ 98.9 mPa · s; the oils’s temperature is kept at 20 ± 0.5 °C to maintain consistent experimental conditions. We note that varying *μ*_o_ in the explored range has a minute effect on the interfacial tension *σ* between the working liquids and the oils^13^. However, *σ* decreases from 35.7 mN · m^−1^ to 25.2 mN · m^−1^ if *μ* increases from 1 mPa · s to 258.8 mPa · s and *μ*_o_ is fixed (Fig. 1, Supplementary Information).

In order to apply a potential difference between a droplet and the ITO layer, we immerse one end of a tungsten wire (18 μm in diameter) in the droplet and connect the other end to the positive electrode of a power supply (IT6723G, ITECH) via a solid-state relay (SSR). The vertical distance between the substrate and the tip of the tungsten wire is roughly equal to the droplet’s radius. The ground electrode of the power supply is connected to the ITO layer. By using the SSR to close the circuit momentarily, we generate an electrical pulse in the form of a step function between the wire and the ITO layer. We observe that the droplet reacts almost immediately after the circuit is closed, suggesting that its electrical response time is very small compared to its hydrodynamical response time^14^. The amplitude of the applied voltage is varied in the range 60 V ≤ *U* ≤ 110 V, which translates to that of electrowetting number as 0.35 ≤ *η* ≤ 1.18. We note that all of our experiment on the transient dynamics is carried out without contact line saturation, which occurs at *U* = 115 V (Fig. 1b). We trigger a high-speed camera (SAX2, Photron) with the generated pulse to record the deformation process of droplets (Fig. 1c). For a set of control parameters *R*, *μ*, and *η*, we repeat the experiment 5 to 7 times and measure the spreading radius *r* as a function of time *t* (Fig. 1d).

**Figure 2:**
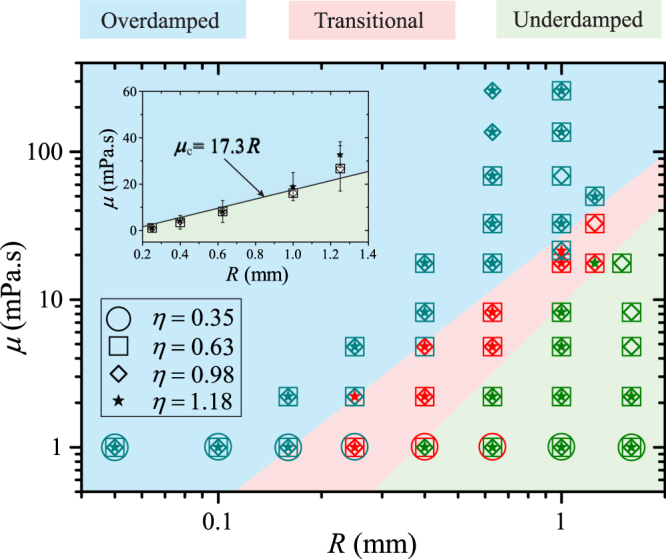
Phase diagram showing different transient dynamics for EWOD actuated droplets. The varying parameters are droplet radius *R*, viscosity *μ*, and electrowetting numbers *η*. The surrounding liquid is silicone oil having viscosity *μ*_o_ = 4.6 mPa · s. The transitional regime includes experiments in which the transient dynamics switch between overdamped and underdamped behaviours. The extent of the transitional regime indicates the experimental uncertainty in determining the overdamped-to-underdamped (O-U) transition. Inset: Phase diagram showing the O-U transition, with error bars representing the extent of the transitional regime. The straight line is a linear fit to the O-U transition.

We observe three characteristic transient behaviours of the spreading radius *r*. For each set of (*R*, *μ*, *η*), we categorise the corresponding behaviour as *overdamped* if *r*(*t*) varies monotonically (Fig. 1d, *μ* = 258.8 mPa · s), and *underdamped* if overshootings are consistently observed in *r*(*t*) (Fig. 1d, *μ* = 1.0 mPa · s). We note that the underdamped behaviour is always accompanied by capillary waves at the liquid-oil interface (see Fig. 1c). In the case that repetitive runs of the same parameters result in alternative characteristics, we categorise the behaviour as *transitional* (Fig. 1d, *μ* = 17.6 mPa · s). In Fig. 2, we show the phase diagram of these behaviours for wide ranges of control parameters: 0.05 mm ≤ *R* ≤ 1.6 mm, 1.0 mPa · s ≤ *μ* ≤ 258.8 mPa · s, and 0.35 ≤ *η* ≤ 1.18. The overdamped and underdamped regimes are clearly separated by the transitional regime. Among the control parameters, *η* has a negligible effect on the transition between these regimes. We attribute the extent of the transitional regime to irregularities in physical and chemical properties of the substrate; such effects could be minimised with better surface treatments. Thus, the overdamped-to-underdamped transition is possible with an uncertainty indicated by the extent of the transitional regime (Fig. 2, inset).

We now examine the transient dynamics in each of the overdamped and underdamped regimes. In both regime, the concentrated charge density along the liquid-oil interface at the vicinity of the three-phase contact line (TCL) causes a net force *F*_el_ = *ησ* that pulls the liquid horizontally^1,15^. We note that this driving force is only applicable for the case of electrowetting actuation without contact line saturation. In the overdamped regime, the dominant factor opposing to the driving force is the contact line friction, which is estimated as *F*_ct_ = *λu*_ct_, where *λ* is the frictional coefficient and *u*_ct_ is the TCL velocity^16,17^. The dimension of *λ* is dynamic viscosity, and those of both *F*_ct_ and *F*_el_ are forces per unit length. If we denote *τ*_o_ the characteristic timescale for the spreading motion in the overdamped regime, the TCL velocity is *u*_ct_ ≈ *r*_e_/*τ*_o_, where *r*_e_ = *βR* is the spreading radius at equilibrium. Here, *β* can be derived based on the assumptions that the shape of a droplet remains spherical at its equilibrium states and its volume is conserved: $\beta ={r}_{{\rm{e}}}/R={\mathrm{(1}-{\cos }^{2}{\theta }_{{\rm{e}}})}^{\mathrm{1/2}}{\mathrm{(4(1}-\cos {\theta }_{{\rm{e}}}{)}^{-2}{\mathrm{(2}+\cos {\theta }_{{\rm{e}}})}^{-1})}^{\mathrm{1/3}}$, where cos *θ*_e_ = cos *θ*_0_ + *η* (Young-Lippmann equation).

**Figure 3:**
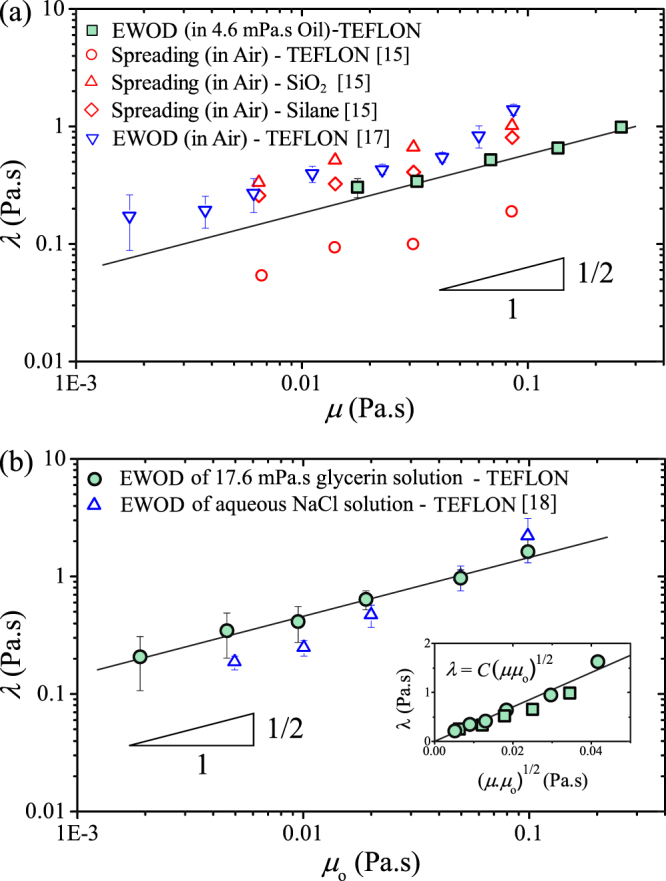
(**a**) Log-log plot of *λ* versus viscosity *μ* of working liquids. Datasets collected in air and on different insulating layers are shown for comparison. The solid line represents the scaling law *λ* ~ *μ*^1/2^. (**b**) Log-log plot of *λ* versus viscosity *μ*_o_ of the surrounding liquids (silicone oils). We only show data in the overdamped regime, i.e., for droplet actuation without capillary waves. The solid line represents the scaling law $\lambda \sim {\mu }_{{\rm{o}}}^{\mathrm{1/2}}$. Inset: Log-log plot of *λ* versus (*μμ*_o_)^1/2^ for our experimental data. The solid line represents the scaling law *λ* = *C*(*μμ*_o_)^1/2^, where *C* = 32.9 ± 3.2 is the fitting parameter.

**Figure 4:**
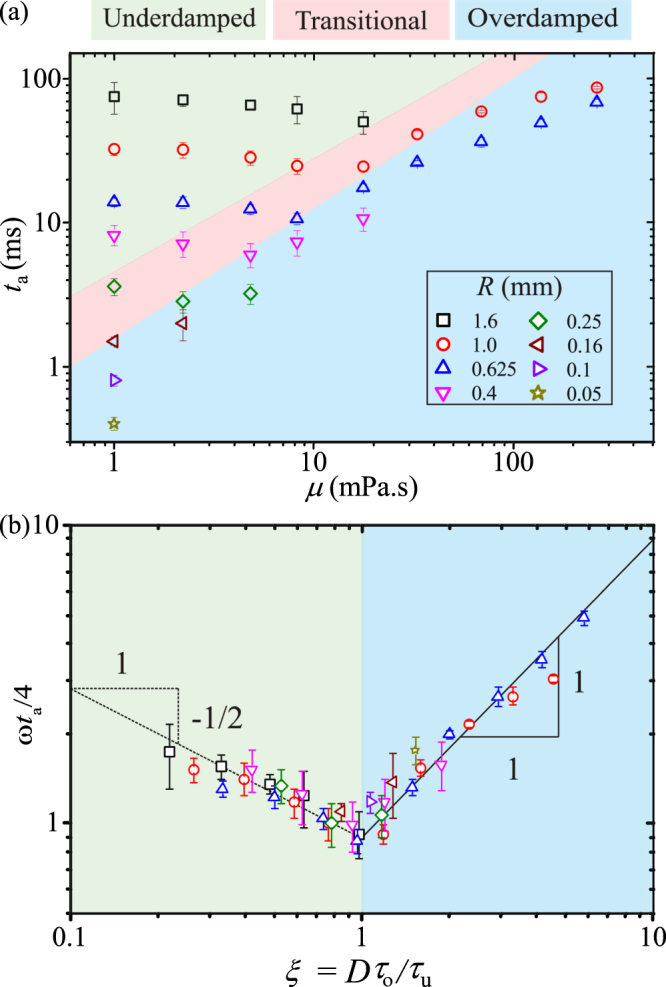
(**a**) Log-log plot of the actuation time *t*_a_ versus *μ* for different droplet size and at *η* = 0.98. The colored areas are indicative of different transient dynamics. (**b**) Log-log plot of *ωt*_a_/4 versus *ξ*. The minimum point *ωt*_a_/4 = 1 occurs at *ξ* = 1.

By balancing the friction with the driving force, we obtain *τ*_o_ = *λr*_e_/*ησ*. The friction coefficient *λ* is a function of both viscosities *μ* and *μ*_o_, and can be measured experimentally (see Methods and Fig. 2, Supplementary Information). In Fig. 3a, we show a log-log plot of the measured values of *λ* versus *μ*. The presented data are consistent with the scaling law *λ* ~ *μ*^1/2^ and other datasets obtained for spreading of glycerol droplets in air and on surfaces coated by Teflon^16,18^, Silicon dioxide (SiO_2_)^16^, and Silane^16^. The vertical shifts between datasets reflect variations in substrate properties and surrounding media. Similarly, the data shown Fig. 3b indicate the scaling law $\lambda \sim {\mu }_{{\rm{o}}}^{\mathrm{1/2}}$, consistent with the data collected for aqueous sodium chloride droplets in silicone oils^13^. Thus, this result suggests *equal* contributions of *μ* and *μ*_o_ to variations in *λ*. In other words, the dependence of *λ* on the bulk viscosities can be described as *λ* = *C*(*μμ*_o_)^1/2^ for the tested ranges of *μ* and *μ*_o_, where *C* is a constant that depends on the roughness and chemical properties of the surface^16^. Indeed, all of our data collapse to a single curve when plotting *λ* versus (*μμ*_o_)^1/2^, as shown in the inset of Fig. 3b. A best fit to the our data gives *C* = 32.9 ± 3.2, a constant specific to the properties of our substrate. Here we emphasise that the scaling law *λ* ~ (*μμ*_o_)^1/2^ is applicable for electrowetting actuation of droplets immersed in silicone oils. An extrapolation of this scaling law to the case in which the outer medium is air (*μ*_o_ = 1.81 × 10^−2^ mPa · s) may require a substantial extension of experimental data towards the lower limit of *μ*_o_ and merit a separate study. We therefore arrive at the expression for the characteristic timescale in the overdamped regime *τ*_o_ = *C*(*μμ*_o_)^1/2^*r*_e_/*ησ*. With this timescale, we obtain an excellent data collapse for the spreading radius *r*(*t*) in the overdamped regime (Fig. 3, Supplementary Information). We note that the viscous time scale *μR*/*σ* cannot be used to collapse our data in the overdamped regime (Fig. 4, Supplementary Information). Thus, the data collapse using *τ*_o_ indicates that *τ*_o_ characterises the transient dynamics in the overdamped regime.

In the underdamped regime, we assume that viscosity is negligible and the driving force is only resisted by the droplet’s inertia. Thus, by balancing the driving force and inertia, one finds that droplets in this regime oscillate with characteristic frequency *ω* = (*ησ*/*ρR*^3^)^1/2^ ^19^. As a result, the characteristic timescale for the droplets to reach maximum deformation is *τ*_u_ = *π*(*ρR*^3^/*ησ*)^1/2^ ^19–21^. We use *τ*_u_ to normalise the data of spreading radius *r*(*t*) in the underdamped regime and observe data collapse for all control parameters (*R*, *η*, *μ*, *μ*_o_) (Fig. 5, Supplementary Information). This strongly suggests that *τ*_u_ is the characteristic time of the underdamped regime.

Let us discuss parameter relations at the overdamped-to-underdamped (O-U) transition. We argue that the characteristic timescales of the two regimes are comparable at the O-U transition. In other words, the condition for the O-U transition to occur is *ξ* = *Dτ*_o_/*τ*_u_ = 1, where *D* is a prefactor of unity order and is independent of the control parameters. Here, the so-called damping ratio *ξ* can be fully expanded as *ξ* = (*DC*/*π*) (*βη*^−1/2^) (*μμ*_o_)^1/2^ (*ρσR*)^−1/2^, revealing similar physical significance as the Ohnesorge number with an additional electrical term *βη*^−1/2^. The damping ratio is used to indicate whether the droplet behaviour is in the underdamped regime (*ξ* < 1), or in the overdamped regime (*ξ* > 1). The condition for the O-U transition to occur, *ξ* = 1, translates to a linear relation between the *critical viscosity μ*_c_, defined as the viscosity at the transition, and the droplet radius *R*:

1 $${\mu }_{{\rm{c}}}={(\frac{\pi }{CD})}^{2}\frac{\eta }{{\beta }^{2}}\frac{\rho \sigma }{{\mu }_{{\rm{o}}}}R.$$

This relation is consistent with the O-U transition shown in the inset of Fig. 2; fitting *μ*_c_ to the data at the transition in the phase diagram gives *D* = 1.49 ± 0.21 for all tested values of the electrowetting numbers *η* (Fig. 2, inset). Moreover, we note that the composite term *ηβ*^−2^ carries the dependence of *μ*_c_ on both the applied voltage and the inherent electrical properties of the system, e.g., the thickness and the dielectric constant of the dielectric layer. For *η* varying from 0.35 to 1.18 in our experiments, the value of *ηβ*^−2^ changes in a narrow range, from 0.55 to 0.62. Thus, we conclude that *μ*_c_ varies linearly with *R* and depends weakly on *η* for the explored ranges of parameters.

To obtain a quantitative description of the actuating motion of droplets under EWOD conditions, we measure the actuation time *t*_a_, defined in practice as the duration for the spreading radius to reach 95% of the radius at equilibrium state after a voltage is applied. As illustrated in Fig. 1d, *t*_a_ depends strongly on the viscosity. More generally, it suggests that *t*_a_ is closely linked to the transient dynamics, i.e., overdamped or underdamped. In Fig. 4a, we show a plot of *t*_a_ versus *μ* for a fixed applied voltage (*η* = 0.98) and various droplet sizes (0.05 mm ≤ *R* ≤ 1.6 mm). Indeed, different transient dynamics result in distinctive behaviours for the actuation time: *t*_*a*_ decreases with *μ* in the underdamped regime, but increases in the overdamped regime. We explore this link, i.e., between *t*_a_ and *μ*, *R*, *η* for different transient dynamics, by making an analogy between an actuating droplet and a mass-spring system. We take *ω* as the natural frequency and the ratio *ξ* = *Dτ*_o_/*τ*_u_ as the damping ratio of the analogous mass-spring system. We note that for a mass-spring system of natural frequency *ω* and damping ratio *ξ*, the actuation time, i.e., the duration for the system to reach an equilibrium state, is specified as *τ*_s_ = 4/*ξω* = (4*π*/*DC*)*ρR*^2^*β*^−1^ (*μμ*_o_)^−1/2^ ^22^. This suggests that *τ*_s_ is the characteristic timescale for the actuating motion of droplets in both transient dynamics. As a result, we nondimensionalise *t*_a_ and *μ* as *t*_a_/*τ*_s_ = *t*_a_*ξω*/4 and *μ*/*μ*_c_ = *ξ*^2^, respectively. The link between *t*_a_ and *μ*, in a power form, is therefore equivalent to that of *ωt*_a_/4 and *ξ*. In the Fig. 4b, we show a log-log plot of *ωt*_a_/4 versus *ξ* for all the data shown in Fig. 4a. In both the underdamped and overdamped regimes, excellent data collapses confirm that *τ*_s_ indeed represents the actuation time of droplets regardless of transient dynamics. In addition, the minimum value of the cloud of data occurs at *ξ* = (*μ*/*μ*_c_)^1/2^ = 1 and *ωt*_a_/4 = 1. We conclude that for fixed values of *R* and *η*, minimisation of *t*_a_ is *always* achieved at *μ* = *μ*_c_. Remarkably, in the underdamped regime the transition time reduces as the damping ratio increases. This indicates that the internal energy transfer from oscillation to spreading is facilitated by increasing the viscosity. In the overdamped regime, the energy dissipation plays an important role, thus leading to the slow-down of spreading with increasing damping ratio. This demonstrates that Kramers’ reaction-rate theory^23^ may be helpful to understand electrowetting dynamics.

## Conclusions

The transient dynamics of actuating droplets exhibit either underdamped or overdamped behaviour; the overdamped-to-underdamped (O-U) transition is insensitive to the applied voltage *U*, but is strongly dependent on the droplet size *R* and liquid viscosity *μ*. This results in a linear relation between the droplet size *R* and the critical viscosity *μ*_c_ at the O-U transition. The droplet actuation time *t*_a_ does not only depend on the system’s control parameters, but also on the transient dynamics: it is minimised at the O-U transition. Interestingly, the weak dependence of the O-U transition on the driving force implies that our analysis may be applicable to the co-planar electrowetting setting^24^ in which the resistive forces remain, but the driving force is modified due to partial coverage of co-planar electrodes on the substrate. Based on similar force analysis, we anticipate that the O-U transition in the top-plate electrowetting setting^25,26^ may be determined by taking into account an additional resistive force: the capillary pressure gradient of the curved liquid surface between the two plates of the top-plate setting. Finally, the transition time shows the cross-over feature from under-to over-damping as nicely described in Kramers’ reaction-rate theory.

## Methods

### Friction coefficient measurement

Suppose that the three-phase contact line (TCL) has velocity *u*_ct_ at time *t* after a voltage is applied. The driving force acting on the TCL is (cos *θ*_e_ − cos *θ*_d_)*σ*, and the frictional force is −*λu*_ct_^17^. Because inertia is neglected in the overdamped regime, a force balance at the TCL gives (cos *θ*_e_ − cos *θ*_d_)*σ* = *λu*_ct_, where *θ*_e_ and *θ*_d_ respectively are the contact angles at the equilibrium state and at time *t*^27^. From the high-speed recordings of the TCL motion, we calculate *λ* based on the measured values of *θ*_e_, *θ*_d_, and *u*_ct_ (Fig. 2, Supplementary Information).

## Electronic supplementary material

Supplementary Information
