# Supplementary material for: Universal Transient Dynamics of Electrowetting Droplets
Source: Sci Rep. 2018 Jan 16;8:836. doi: 10.1038/s41598-018-19167-7 (PMC5770462; doi:10.1038/s41598-018-19167-7)
Supplement: Supplementary file 1 — Supplementary Information [file 41598_2018_19167_MOESM1_ESM.pdf]

Universal Transient Dynamics of Electrowetting Droplets: Supplementary Information

Quoc Vo[1], Haibin Su[2], and Tuan Tran[1,*]

[1]Mechanical and Aerospace Engineering School, Nanyang Technological University, 50 Nanyang Avenue, 639798, Singapore.
[2]Institute of Advanced Studies, Nanyang Technological University, 60 Nanyang View, 639673, Singapore
[*]ttran@ntu.edu.sg

ABSTRACT

Droplet spreading on substrates by electrowetting exhibits either of the two transient behaviours: one characterised by contact line oscillation, and the other one by slow spreading dynamics. The transition between these behaviours remains elusive due to the current limited understanding of the spreading dynamics on the hydrodynamical and electrical properties of electrowetting systems. To understand this transition we propose a model capturing the transition's occurrence based on both the hydrodynamical and electrical parameters. We derive the critical viscosity at which the transition occurs and reveal its subtle and often hidden dependence on the electrowetting dynamics. We find and experimentally verify that the condition for minimization of droplets' actuation time is only achieved at the transition. Particularly, the transition time as a function of damping ratio exhibits the general feature of Kramers' reaction-rate theory.

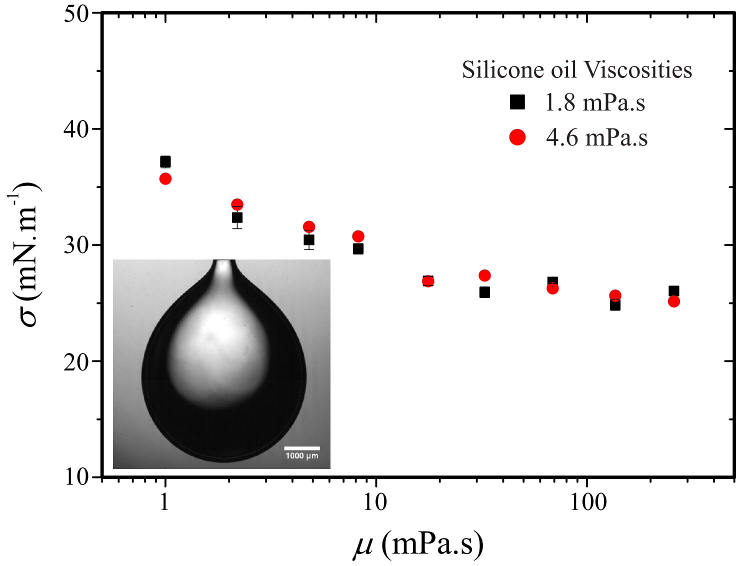

Figure 1. Log-linear correlation between the interfacial tension σ and the viscosity μ of the working fluids (glycerin solutions) as the mass concentration of glycerin varies from 0 % to 91 %. There are two sets of data corresponding to two values of viscosity μ_o of the outer fluid (silicone oil). We measure the interfacial tension using the pendant drop method (inset). The measured values of σ change from $35.7\,\mathrm{mN \cdot m^{-1}}$ to $25.2\,\mathrm{mN \cdot m^{-1}}$, while μ increases from $1\,\mathrm{mPa \cdot s}$ to $258.8\,\mathrm{mPa \cdot s}$ as the mass concentration of glycerin varies from 0% to 91 %, consistent with reported values for similar solutions[1,2].

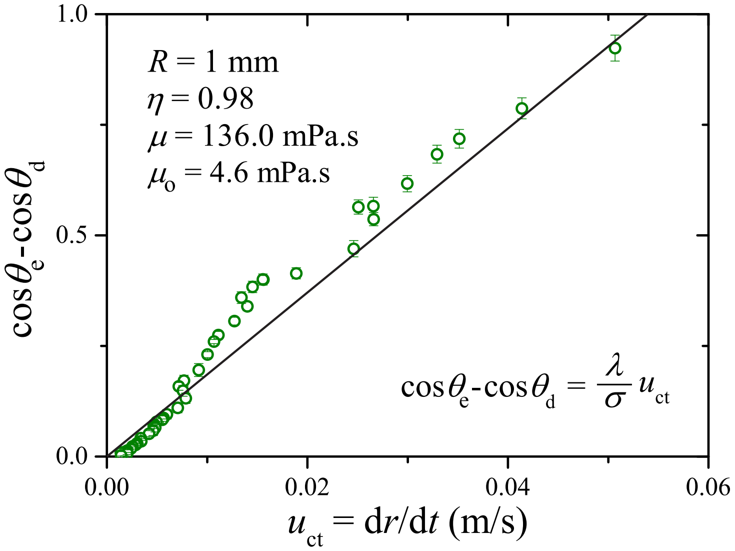

Figure 2. Representative plot of $\cos\theta_e - \cos\theta_d$ versus u_{ct} for a droplet of diameter 1 mm and liquid viscosity 136 mPa·s spreading on TEFLON surface under the applied voltage 100 V. The friction coefficient λ is calculated using the slope λ/σ of the linear fit to the data. In the plot, $\lambda/\sigma = 18.65 \pm 1.34$, giving $\lambda = 0.653 \pm 0.05$.

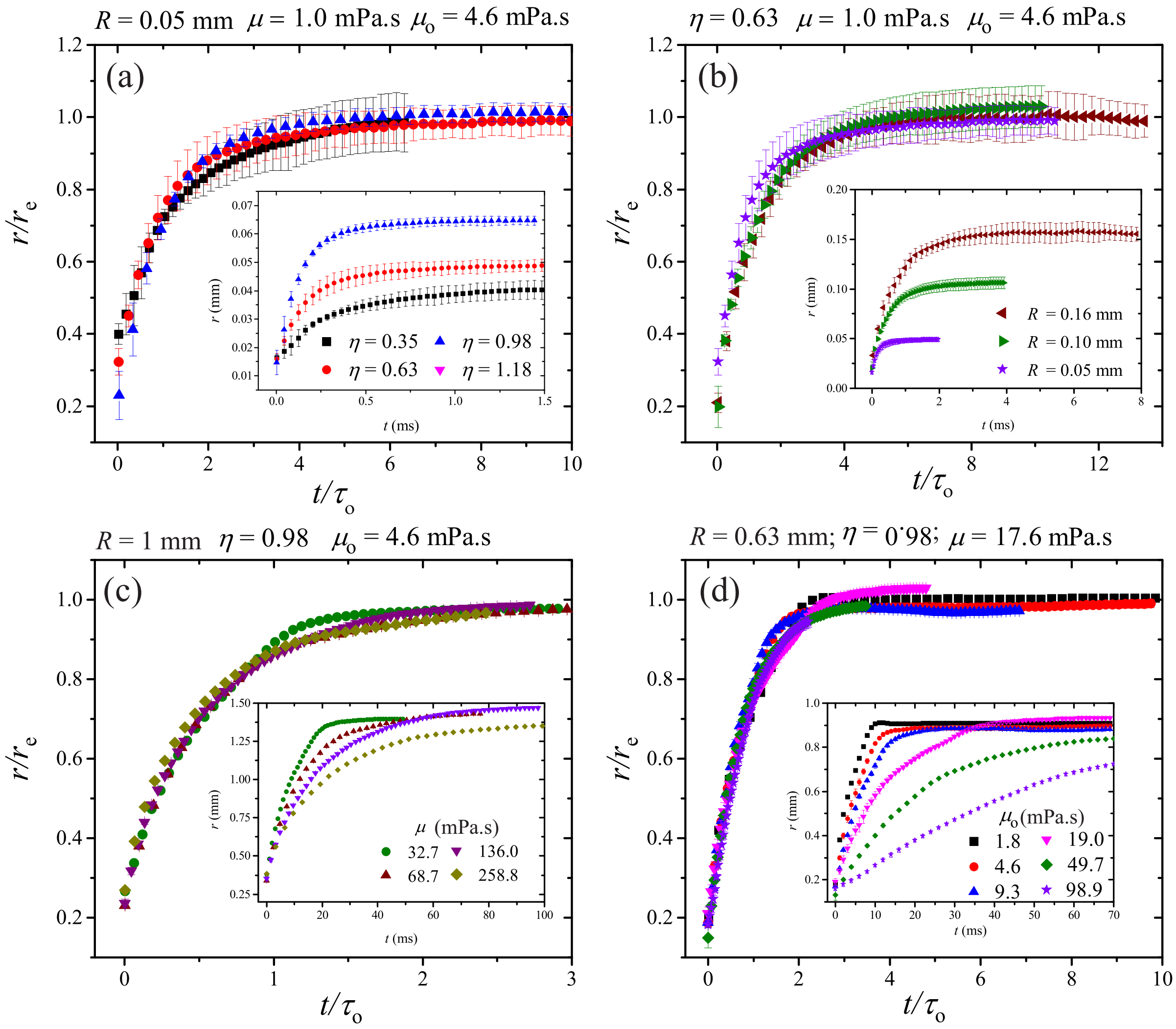

Figure 3. Plots of normalized spreading radius r/r_e versus normalized time t/τ_o in the *overdamped* regime for four varying control factors: (a) - Electrowetting number η, (b) - Droplet size R, (c) - Liquid viscosity μ, (d) - Silicone oil viscosity μ_o. Insets showing plots of r versus t for the corresponding normalized data in the main plots. Experimental conditions and properties of fluids are given in each plot.

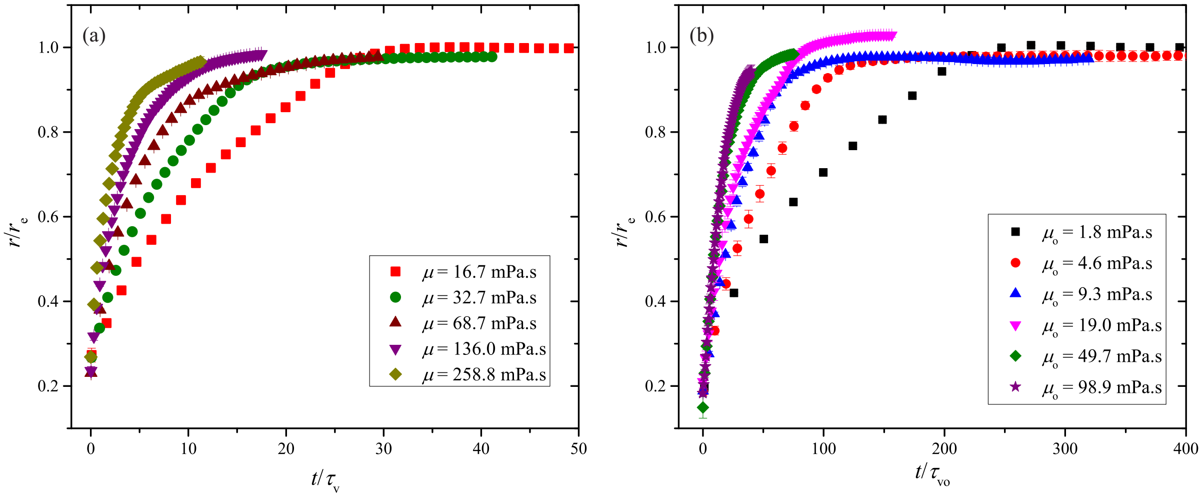

Figure 4. (a) Normalised spreading radius r/r_e vs. spreading time normalised by the viscous time scales $\tau_\mathrm{v} = \mu R/\sigma$ for data of different μ, and (b) normalised spreading radius r/r_e vs. spreading time normalised by the viscous time scales $\tau_\mathrm{vo} = \mu_\mathrm{o} R/\sigma$ for data of different μ_o. The data are not collapsed, indicating that the both viscous time scales τ_v and τ_vo cannot be used to characterise the spreading dynamics in the overdamped regime.

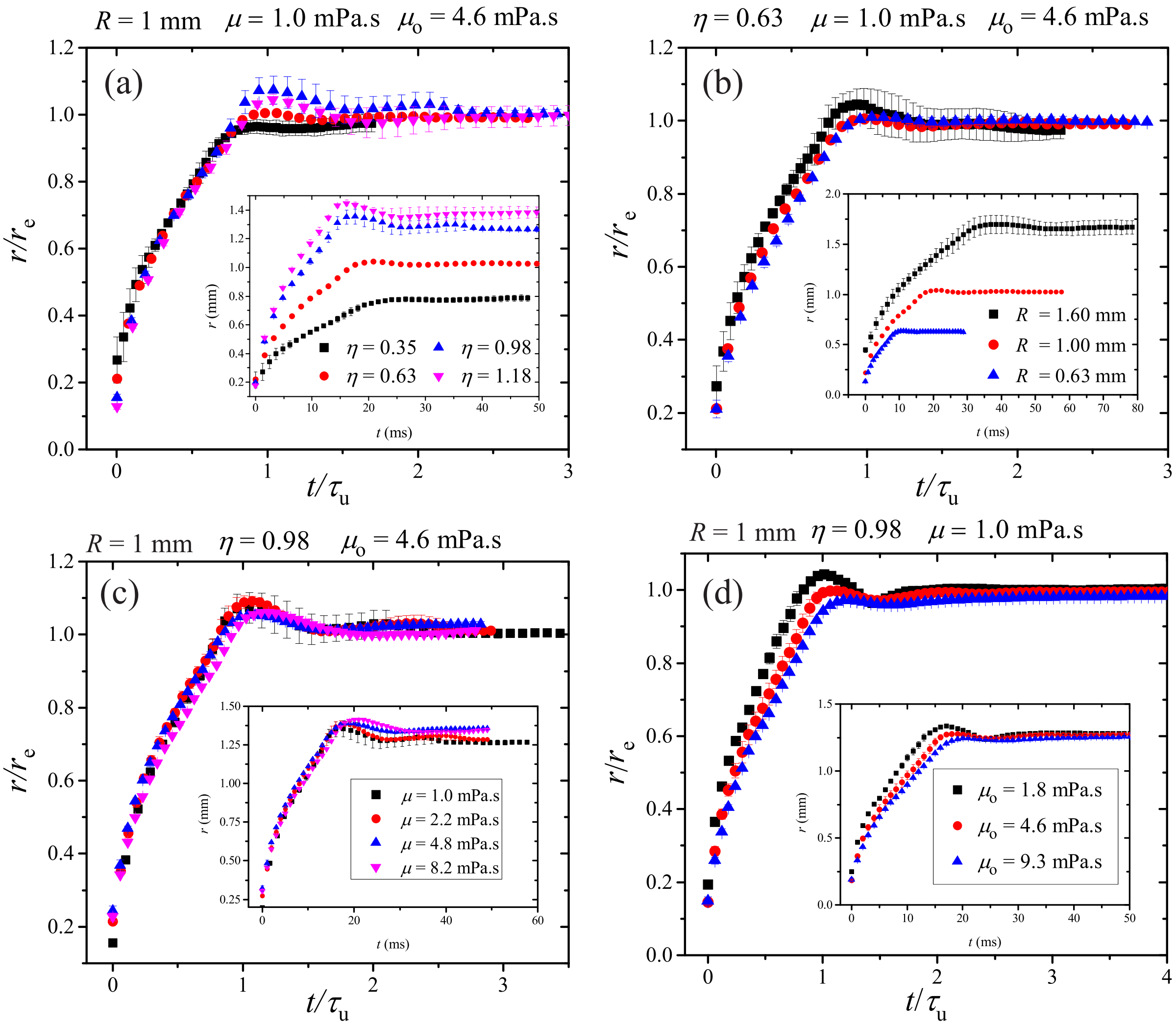

Figure 5. Plots of normalized spreading radius r/r_e versus normalized time t/τ_u in the *underdamped* regime for four varying control factors: (a) - Electrowetting number η, (b) - Droplet size R, (c) - Liquid viscosity μ, (d) - Silicone oil viscosity μ_o. Insets showing plots of r versus t for the corresponding normalized data in the main plots. Experimental conditions and properties of fluids are given in each plot.

References

1. Peters, F., Arabali, D. Interfacial tension between oil and water measured with a modified contour method. *Colloids Surfaces A Physicochem. Eng. Asp.* **426**, 1-5, (2013).

2. Banpurkar, A., G., Nichols, K., P., and Mugele, F. Electrowetting-Based Microdrop Tensiometer. *Langmuir* **24**, 10549-10551 (2008).

3. Hong, J., Kim, Y. K., Kang, K. H., Oh, J.,M., and Kang, I. S. Effects of drop size and viscosity on spreading dynamics in dc electrowetting. *Langmuir* **29**, 9118-9125 (2013).
